# Normal Non-HDL Cholesterol, Low Total Cholesterol, and HDL Cholesterol Levels in Sickle Cell Disease Patients in the Steady State: A Case-Control Study of Tema Metropolis

**DOI:** 10.1155/2016/7650530

**Published:** 2016-12-18

**Authors:** Richard K. D. Ephraim, Patrick Adu, Edem Ake, Hope Agbodzakey, Prince Adoba, Obed Cudjoe, Clement Agoni

**Affiliations:** ^1^Department of Medical Laboratory Science, School of Allied Health Sciences, College of Health and Allied Sciences, University of Cape Coast, Cape Coast, Ghana; ^2^Department of Molecular Medicine, School of Medical Sciences, College of Health, KNUST, Kumasi, Ghana; ^3^Department of Microbiology, School of Medical Sciences, College of Health and Allied Sciences, University of Cape Coast, Cape Coast, Ghana

## Abstract

*Background.* Abnormal lipid homeostasis in sickle cell disease (SCD) is characterized by defects in plasma and erythrocyte lipids and may increase the risk of cardiovascular disease. This study assessed the lipid profile and non-HDL cholesterol level of SCD patients.* Methods.* A hospital-based cross-sectional study was conducted in 50 SCD patients, in the steady state, aged 8–28 years, attending the SCD clinic, and 50 healthy volunteers between the ages of 8–38 years. Serum lipids were determined by enzymatic methods and non-HDL cholesterol calculated by this formula: non-HDL-C = TC-HDL-C.* Results.* Total cholesterol (TC) (*p* = 0.001) and high-density lipoprotein cholesterol (HDL-C) (*p* < 0.0001) were significantly decreased in cases compared to controls. The levels of non-HDL-C, low-density lipoprotein cholesterol (LDL-C), and triglyceride (TG) were similar among the participants. The levels of decrease in TC and HDL were associated with whether a patient was SCD-SS or SCD-SC. Systolic blood pressure and diastolic blood pressure were each significantly associated with increased VLDL [SBP, *p* = 0.01, OR: 0.74 (CI: 0.6–0.93); DBP, *p* = 0.023, OR: 1.45 (CI: 1.05–2.0)].* Conclusion.* Dyslipidemia is common among participants in this study. It was more pronounced in the SCD-SS than in SCD-SC. This dyslipidemia was associated with high VLDL as well as increased SBP and DBP.

## 1. Background

Sickle cell disease (SCD) is a genetic disorder caused by the substitution of valine for glutamic acid at the sixth position of the amino acid *β*-chain of the haem molecule [1, 2] and is characterized by the possession of sickle haemoglobin [3]. It is a significant cause of morbidity and mortality among black individuals and descendants of Negroid race [4]. Life expectancy is shortened with studies reporting average life expectancy of 42 and 48 years for males and females, respectively [5].

SCD is characterized by defect in plasma and erythrocyte lipids associated with chronic oxidative stress [6]. These two morbid processes disturb lipid homeostasis which in turn may lead to atherosclerosis in these patients [7, 8]. Abnormal lipid homeostasis, as well as other haematological disorders, has been reported in SCA and this has been suggested to have the potential to alter membrane fluidity and functions of red blood cells (RBC) in individuals with SCD [9–11].

Mostly, a standard lipid profile (triglyceride, total cholesterol, high-density lipoprotein cholesterol, and low-density lipoprotein cholesterol) is used to assess the risk of coronary artery disease (CAD). Earlier studies in patients with SCD recorded a significant increase in triglyceride (TG) levels and decreased levels of total cholesterol (TC), high-density lipoprotein cholesterol (HDL-C), and low-density lipoprotein cholesterol (LDC-C) [9]. This remains a cause for concern as it is associated with increased mortality [10].

Atherosclerosis, often associated with CAD, is characterized by elevated levels of cholesterol and LDL-C [12]. Pulmonary hypertension, the main form of cardiovascular dysfunction in SCD, is often characterized by low levels of TC and LDL-C. Relying on LDL-C levels to assess cardiovascular disease may be misleading as reported in a recent meta-analysis [11]. That study recorded low levels of LDL-C but high levels of non-HDL-C (TC-HDL-C) in people with cardiovascular disease [11, 13]. It is imperative that these reported dyslipidemia cases in SCD should be interrogated in light of the new findings. In addition, there is a paucity of data on lipid profile in SCD patients in Ghana. In light of the above, we sought to determine the lipid profile and non-HDL-C levels in SCD patients in the steady state.

## 2. Methods

### 2.1. Setting/Design

This hospital-based cross-sectional study was carried out at the sickle cell unit of Tema General Hospital in the Greater Accra region of Ghana.

### 2.2. Participants

A total of 50 SCD patients (12 with HbS and 38 with HbSC haemoglobin variants) were recruited for the study. A total number of 50 healthy, age- and sex-matched controls also participated in the study.

### 2.3. Exclusion/Inclusion Criteria

Patients who have been diagnosed with the sickle cell disease, those with the genotype HbSS and HbSC who are in the steady state, and those who allowed parental consent were included in the study. Exclusion criteria included patients with inflammatory episodes, patients with sickle cell trait, patients on medications that affect lipid metabolism, and those who have had blood transfusion four (4) months prior to the study.

### 2.4. Ethical Consideration

All protocols for the study were approved by the Institutional Review Board (IRB) of the University of Cape Coast as well as the sickle cell clinic of the Tema General Hospital. Participation was voluntary and involved only Ghanaians. Written informed consent was obtained from each participant. All data was deidentified before analysis.

### 2.5. Sampling

In all subjects, 5 mL of overnight fasting venous samples was collected from all eligible subjects: 3 mL was put in plain tube, allowed to clot, and centrifuged at 2500 rpm for 5 minutes and the serum was used for estimation of lipid profile; 2 mL was put in EDTA tube and used for confirmation of their haemoglobin phenotype by cellulose acetate electrophoresis.

### 2.6. Testing

TC and TG concentrations were analyzed by enzymatic assay, whereas HDL-C was estimated calorimetrically. The calculation of VLDL-C was done by VLDL-C = triglyceride/2.2 and LDL-C calculation was done by the following Friedewald equation: LDL-C = TC-HDL-C − (TG/2.2) [14]. Non-HDL-C was calculated by TC-HDL-C [15].

### 2.7. Statistical Analysis

Data was entered into Microsoft Excel (Microsoft, Redmond, WA, USA) and analyzed with SPSS version 16.0 (SPSS Inc., Chicago, IL, USA). The results were expressed as mean ± standard deviation and *t*-test was used to calculate the level of significance. A *p* value of ≤ 0.05 was considered statistically significant. Multivariate logistic regression was done to determine the independent factors of dyslipidemia in SCD.

## 3. Results

Table 1 presents the baseline characteristics of the participants. The mean ages of the sickle cell disease (SCD) patients and those without SCD were 18.14 ± 4.63 years and 21.42 ± 7.76 years, respectively. SCD was found to be more prevalent in females (78.0%) than in males (22.0%), with majority of the SCD patients, 33 (66.0%), aged between 10 and 19 years. Assessments of obesity using BMI was significantly (= 0.002) lower in the SCD patients than in those with no SCD (Table 1). Both systolic and diastolic blood pressure parameters of the SCD patients were significantly lower (<0.0001) compared to healthy subjects. Serum lipid profile showed no statistically significant difference between the two groups as TG, LDL, VLDL, and non-HDL were compared (*p* > 0.05) except for TC and HDL (*p* = 0.001, <0.0001, resp.) (Table 1).

The plasma lipid concentrations, BMI, and blood pressure of the three groups are shown in Table 2. Mean BMI, SBP, and DBP were significantly lower in SC and SS patients (*p* < 0.05) compared to the healthy controls (AA).

TC and HDL cholesterol were significantly lower in SC and SS patients compared to the control groups (*p* < 0.05), despite being higher in the sickle cell patients with SS genotype than in those with SC. In addition, TG/HDL ratio was significantly higher in SC patients than in the healthy controls and SS patients.

Table 3 shows the factors associated with dyslipidemia in SCD patients. Both SBP and DBP were each significantly associated with VLDL [SBP, *p* = 0.01, OR: 0.74 (CI: 0.6–0.93); DBP, *p* = 0.023, OR: 1.45 (CI 1.05–2.0)].

Our study also investigated the relationship of BMI and blood pressure variables with serum lipid profile in SCD patients and healthy individuals. Body mass index showed a nonsignificant inverse correlation with TC, TG, and VLDL in both SCD and healthy patients (see Supplementary Data S1 in Supplementary Material available online at http://dx.doi.org/10.1155/2016/7650530). However, BMI correlation with LDL was negative for SCD patients and positive for healthy controls and vice versa for HDL despite being significant for SCD patients (Supplementary Data S1). SBP was directly related to all lipid profile parameters in the controls with the exception of LDL whereas TG and VLDL showed inverse correlation with SBP in the cases. On the other hand, with the exception of HDL, DBP was positive but not significantly related to TC, TG, LDL, and VLDL in cases whereas in the controls all the lipid profile parameters with the exception of VLDL and non-HDL-C showed a nonsignificant positive correlation with DBP.

## 4. Discussion

This study sought to assess the lipid profile and non-HDL-C levels of sickle cell disease patients in the steady state compared to healthy controls. Our findings showed that cholesterol (total cholesterol, HDL) levels decreased in SCD patients and were dependent on whether the patient has haemoglobin SS or SC; non-HDL remained unchanged between the two groups [16].

Non-HDL-C is a significant predictor of cardiovascular disease among diabetes patients [17]. We observed no significant difference in non-HDL-C levels among our participants. This to our knowledge is the first report on non-HDL-C levels in SCD and thus gives credence to the established evidence that cardiovascular disease in sickle cell disease is mostly due to pulmonary hypertension and not atherosclerosis associated with elevated levels of TC, HDL, and LDL [9, 18, 19].

The decreased TC and HDL in SCD are well documented in almost all the studies that have examined lipids in patients with SCD [20–22]. Hypocholesterolemia in SCD has been attributed to increased erythropoiesis in response to the anaemia associated with SCD as stated in earlier studies [10, 23]. Hypocholesterolemia has been identified as a potential biomarker of the clinical severity of SCD [24].

Consistent with some but not all studies, we recorded low HDL in SCD patients compared to controls [25]. Several reasons including small sample sizes, differences in gender, age, and weight, and variations in disease severity have been ascribed for these inconsistencies [26]. In the general population, low HDL is a recognized risk factor of cardiovascular disease but low HDL also remains a common feature of pulmonary hypertension which is the main cardiovascular disorder associated with SCD [27]. SCD patients with low HDL are more likely to have received more blood transfusions, an indication of the severity of the patient's condition [28].

TG and LDL levels were not significantly different among our participants. The observed TG level is consistent with the findings of Reaven [29] in SCD patients in Nigeria. However, we cannot proffer any reasons for the observed levels of LDL which seems to be at variance with what is recorded in most studies [24, 29] except to say that the use of steady state patients could account for this observation.

The TG/HDL-C ratio also known as the atherogenic index has been implicated in endothelial dysfunction associated with insulin resistance [30]. The increase in this index as observed in this study suggests an increased risk of pulmonary hypertension among our participants [31]. Also, we noted that the TG/HDL-C ratio was higher in SC-SCD patients than in SS-SCD patients, further providing evidence of the high level of TG in the SCD participants. Multivariate analysis showed an association between elevated VLDL levels and blood pressure (BP) with SCD patients being more liable to developing diastolic dysfunction (depicted here by elevated DBP) which ultimately increases mortality [31]. The observed VLDL level is strengthened by the earlier observation of the positive correlation between TG and VLDL. The role of TG in the development of pulmonary hypertension in SCD is well elucidated with high VLDL levels similar to the one recorded further buttressing the TG levels recorded in this study.

## 5. Conclusion

It was evident from this study that dyslipidemia characterized by low HDL and TC was present among the SCD patients who participated this study. It was more pronounced in the SCD-SS than in SCD-SC. Non-HDL levels were unchanged in cases compared to controls. Dyslipidemia especially high VLDL is associated with increased SBP and decreased DBP.

## Supplementary Material

Supplementary data S1 summarizes the correlation between the haemodynamic parameters and lipid profile parameters.

## Figures and Tables

**Table 1:** Demographics and clinical characteristics of participants.

Variable	Cases	Controls	*p* value
	(*n* = 50)	(*n* = 50)	
*Age (years)*	18.14 ± 4.63	21.42 ± 7.76	**0**.**060**
*Gender*			0.488
Male	11 (22.0)	14 (28.0)	
Female	39 (78.0)	36 (72.0)	
*Age group, n (%)*			0.056
<10	2 (4.0)	2 (4.0)	
10–19	33 (66.0)	20 (20.0)	
20–29	15 (30.0)	19 (38.0)	
30–39	0 (0.0)	9 (18.0)	
*BMI (kg/m* ^*2*^)	20.67 ± 3.46	23.22 ± 4.50	**0**.**002**
*BMI, n (%)*			0.077
Underweight	15 (30.0)	9 (18.0)	
Normal	30 (60.0)	26 (52.0)	
Overweight	4 (8.0)	11 (222.0)	
Obese	1 (2.0)	4 (8.0)	
*Blood pressure (mmHg)*			
SBP	108.90 ± 8.13	115.52 ± 5.17	<**0**.**0001**
DBP	71.92 ± 6.88	76.98 ± 5.98	<**0**.**0001**
*Lipid profile*			
TC (mmol/L)	3.62 ± 0.78	4.20 ± 0.98	**0**.**001**
TG (mmol/L)	0.98 ± 0.67	0.97 ± 0.41	0.967
LDL-C (mmol/L)	2.15 ± 0.71	4.27 ± 2.02	0.299
VLDL-C (mmol/L)	0.58 ± 0.08	0.44 ± 0.03	0.108
HDL-C (mmol/L)	1.03 ± 0.33	1.50 ± 0.47	<**0**.**0001**
Non-HDL-C (mmol/L)	2.62 ± 0.68	2.68 ± 1.06	0.738
TG/HDL ratio	1.48 ± 0.47	0.71 ± 0.36	0.109

SBP: systolic blood pressure; DBP: diastolic blood pressure; TC: total cholesterol; TG: triglyceride; LDL-C: low-density lipoprotein cholesterol; VLDL-C: very-low-density lipoprotein cholesterol; HDL-C: high-density lipoprotein cholesterol.

**Table 2:** BMI and lipid profiles in participants stratified by the type of haemoglobin variants.

Parameter	Sickling phenotype			*p* value
	AA	SC	SS	
	(*n* = 50)	(*n* = 12)	(*n* = 38)	
*BMI (kg/m* ^*2*^)	23.21 ± 4.50	20.48 ± 4.18^*∗*^	20.73 ± 3.26^*∗*^	**0**.**009**
*BMI, n (%)*				0.181
Underweight	9 (18.0)	4 (33.3)	11 (28.9)	
Normal	26 (52.0)	7 (58.3)	23 (60.5)	
Overweight	11 (22.0)	0 (0.0)	4 (10.5)	
Obese	4 (8.0)	1 (8.3)	0 (0.0)	
*Blood pressure (mmHg)*				
SBP	115.52 ± 5.17	107.92 ± 7.22^*∗*^	109.21 ± 8.47^*∗*^	<**0**.**0001**
DBP	76.98 ± 5.98	70.42 ± 6.86^*∗*^	72.39 ± 6.91^*∗*^	**0**.**001**
*Lipid profile*				
TC (mmol/L)	4.20 ± 0.98	3.23 ± 0.81^*∗*^	3.74 ± 0.75^*∗*^	**0**.**001**
TG (mmol/L)	0.97 ± 0.41	0.99 ± 0.81	0.97 ± 0.63	0.989
LDL-C (mmol/L)	4.27 ± 2.02	1.86 ± 0.76	2.25 ± 0.68	0.581
VLDL-C (mmol/L)	0.44 ± 0.03	0.64 ± 0.20	0.57 ± 0.09	0.241
HDL-C (mmol/L)	1.50 ± 0.47	0.93 ± 0.31^*∗*^	1.06 ± 0.33^*∗*^	<**0**.**0001**
Non-HDL-C (mmol/L)	2.68 ± 1.06	2.38 ± 0.78	2.70 ± 0.65	0.533
TG/HDL ratio	0.71 ± 0.36	2.71 ± 1.91^*∗*^	1.10 ± 0.19^+^	0.035

^*∗*^Significantly different from the control group (AA) (*p* < 0.05). ^+^Significantly different from the group (SC) (*p* < 0.05).

**Table 3:** Factors associated with high TC, TG, and VLDL and low HDL in sickle cell disease patients.

Parameters	TC ≥ 5.0 mmol/L	*p* value	TG ≥ 1.70 mmol/L	*p* value	VLDL ≥ 1.04	*p* value	HDL < 1.10 mmol/L	*p* value
	OR (95% CI)		OR (95% CI)		OR (95% CI)		OR (95% CI)	
Age (years)	1.12 (0.62–1.67)	0.943	1.03 (0.77–1.38)	0.837	1.21 (0.95–1.52)	0.119	0.97 (0.85–1.12)	0.696
BMI (kg/m^2^)	0.71 (0.30–1.71)	0.444	0.80 (0.52–1.23)	0.309	0.75 (0.50–1.12)	0.154	0.89 (0.73–1.07)	0.200
SBP	95.46 (—)	0.993	0.85 (0.69–1.06)	0.154	0.74 (0.60–0.93)	**0**.**010**	0.87 (0.76–0.98)	0.260
DBP	0.68 (0.14–3.19)	0.621	1.26 (0.91–1.74)	0.166	1.45 (1.05–2.00)	**0**.**023**	1.12 (0.97–1.29)	0.130
