# Supplementary material for: Normal Non-HDL Cholesterol, Low Total Cholesterol, and HDL Cholesterol Levels in Sickle Cell Disease Patients in the Steady State: A Case-Control Study of Tema Metropolis
Source: J Lipids. 2016 Dec 18;2016:7650530. doi: 10.1155/2016/7650530 (PMC5203913; doi:10.1155/2016/7650530)
Supplement: Supplementary file 1 — Supplementary data S1 summarizes the correlation between the haemodynamic parameters and lipid profile parameters. [file 7650530.f1.pdf]

Supplementary Data S1

Correlation of Age, BMI, and Blood pressure variables to serum lipid profile among sickle cell disease (Upper portion) and healthy individuals (Lower Portion).

**Parameter**		**AGE**	**BMI**	**SBP**	**DBP**	**TC**	**TG**	**LDL**	**VLDL**	**HDL**	**Non-HDL**	**TG/HDL**
AGE	R	1	-0.072	0.225	-0.028	0.002	-0.083	-0.085	0.078	0.251	-0.125	-0.184
	P-value		0.621	0.117	0.848	0.991	0.565	0.556	0.591	0.078	0.385	0.201
BMI	R	-0.132	1	-0.145	-0.153	-0.218	-0.155	-0.256	-0.185	0.150	-.353*	-0.175
	P-value	0.360		0.315	0.288	0.128	0.283	0.073	0.197	0.300	0.012	0.225
SBP	R	-0.090	0.138	1	.719**	0.186	-0.074	0.150	-0.147	0.199	0.156	-0.04
	P-value	0.532	0.34		0.000	0.196	0.61	0.300	0.309	0.166	0.279	0.782
DBP	R	0.009	0.112	0.056	1	0.114	0.096	0.103	0.12	-0.029	0.186	0.157
	P-value	0.951	0.439	0.697		0.429	0.507	0.475	0.408	0.842	0.196	0.276
TC	R	0.196	-0.257	0.055	0.009	1	0.075	.871**	-0.099	.496**	.875**	-0.059
	P-value	0.173	0.071	0.706	0.949		0.604	0.000	0.495	0.000	0.000	0.684
TG	R	0.108	-0.187	0.161	0.005	.404**	1	-0.243	.364**	-0.227	0.213	.711**
	P-value	0.454	0.193	0.264	0.975	0.004		0.09	0.009	0.113	0.138	0.000
LDL	R	0.117	0.006	-0.019	0.067	-0.138	0.004	1	-0.186	0.22	.865**	-0.134
	P-value	0.417	0.966	0.897	0.642	0.341	0.978		0.197	0.125	0.000	0.354
VLDL	R	0.110	-0.193	0.159	-0.004	.407**	.999**	0	1	-0.181	-0.028	.300*
	P-value	0.445	0.179	0.271	0.98	0.003	0	0.998		0.209	0.846	0.034
HDL	R	-0.013	-0.127	0.126	0.001	0.156	0.005	-0.044	0.001	1	0.096	-.508**
	P-value	0.930	0.379	0.382	0.993	0.279	0.974	0.763	0.996		0.506	0.000
Non-HDL	R	0.153	-0.18	0.001	-0.025	.899**	.386**	-0.101	.391**	-0.258	1	0.174
	P-value	0.288	0.212	0.995	0.864	0.000	0.006	0.485	0.005	0.07		0.228
TG/HDL	R	0.053	-0.04	0.141	0.082	0.163	.791**	-0.001	.792**	-.543**	.378**	1
	P-value	0.715	0.781	0.328	0.571	0.258	0.000	0.997	0.000	0.000	0.007	
